# Antigen-specific antibody and polyfunctional T cells generated by respiratory immunization with protective *BurkholderiaΔtonBΔhcp1* live attenuated vaccines

**DOI:** 10.1038/s41541-021-00333-4

**Published:** 2021-05-13

**Authors:** Nittaya Khakhum, Preeti Bharaj, David H. Walker, Alfredo G. Torres, Janice J. Endsley

**Affiliations:** 1 Department of Microbiology and Immunology, University of Texas Medical Branch, Galveston, TX USA; 2 Department of Pathology, University of Texas Medical Branch, Galveston, TX USA

**Keywords:** Live attenuated vaccines, Humoral immunity

## Abstract

Melioidosis, caused by *Burkholderia pseudomallei* (*Bpm*), lacks a vaccine. We identify the immune correlates of protection induced by *B. mallei* Δ*tonB* Δ*hcp1* (CLH001) and *Bpm* Δ*tonB* Δ*hcp1 (*PBK001) vaccines against inhalational melioidosis. Mucosal immunization with either vaccine generates *Bpm*-specific IgM and IgG (IgG_2__b/c _> IgG_1_ > IgG_3_) antibodies in sera and lungs, and lung IgA antibodies. Sera confers complement-independent bactericidal activity and macrophages opsonophagocytic uptake but is insufficient in passive transfer experiments to provide significant protection. Both vaccines elicit memory Th1 and Th17 CD4^+^ T-cell responses in lung and spleen after *Bpm* antigen-specific recall. The PBK001 vaccine is superior in generating respiratory IgA post-boost, anamnestic IgG at challenge, T-cell recall to specific antigen, and development of diverse polyfunctional memory T-cell pools. Analysis of lung histology suggests that potent polyfunctional T-cell memory and/or IL-17 signatures generated with PBK001 vaccination may be associated with moderate lung inflammation post vaccination.

## Introduction

The pathogenic Gram-negative bacterium*, Burkholderia pseudomallei* (*Bpm*) is the etiologic agent of the disease melioidosis, occurring in both humans and animals^1^. The organism is a saprophyte that resides in the soil, water, and plants, primarily in tropical and subtropical regions of northern Australia and parts of Southeast Asia and the Indian peninsula^2–4^. However, recent environmental suitability analysis indicated that the highest risk zones to encounter *Bpm* are Southeast and South Asia, tropical Australia, Western sub-Saharan Africa, and South America. Current modeling studies estimate that 165,000 cases of melioidosis resulting in 89,000 deaths occur worldwide annually^5^. Clinical melioidosis cases can occur due to acute infections (85%) that frequently include sepsis; as well as chronic (11%) and reactivation of latent infections (4%)^6^. The predominant mode of transmission of melioidosis is percutaneous inoculation, followed by inhalation (aerosol), ingestion (contaminated water), and rare reports of other routes of infection (vertical, zoonotic, and sexual intercourse)^6,7^. The risk factors for melioidosis include sex (male), diabetes mellitus, alcohol consumption, immune compromise, and presence of chronic disease (renal and pulmonary)^4^. Delayed treatments due to frequent misdiagnosis and lack of an available and approved vaccine against melioidosis are a public health concern with this underreported disease.

Due to the current burden of disease and its biothreat potential, the development of effective vaccines for melioidosis is urgent^8^. Like many other intracellular pathogens, *Bpm* is able to survive in phagocytic cells, including macrophages, neutrophils, and monocytes^9,10^, avoiding the induction of protective immune responses^11^. The intracellular nature of *Bpm* strongly suggests that both humoral and cellular immunity are required to induce complete protection^11,12^. In previous studies, we have successfully developed live attenuated vaccines from *B. mallei* (named CLH001) and *B. pseudomallei* (named PBK001) by deleting the *tonB* and *hcp1* genes^13–15^. The construction of these double deletion mutants eliminates the possibility of reversion to wild-type virulent phenotype, which is a safety concern for live attenuated strains.

Recently, CLH001 and PBK001 have been approved by the US Federal Select Agent Program to be excluded from the select agent register and can be handled under biosafety level 2 (BSL-2) conditions. Both CLH001 and PBK001 are attenuated, safe, and unable to persist in vaccinated animals. Intranasal (i.n.) vaccination with CLH001 and PBK001 has been demonstrated to provide 87.5 and 100% protection, respectively, against aerosolized *Bpm* infection in the C57BL/6 mouse model of melioidosis^13,14^. Additionally, up to 70% of mice receiving CLH001 or PBK001 showed bacterial clearance from lung and other target organs, strongly indicative of development of sterilizing immunity in most of the vaccinated animals^13,14^. Live attenuated vaccines, including those described here, have shown promise for inducing effective immunity against melioidosis^13–19^. Similar to other melioidosis vaccine candidates^16–19^, the immune basis for the potent efficacy of the CLH001 and PBK001 vaccines is not fully understood.

In the present study, we conducted a thorough interrogation of the development of humoral and cellular immune responses following i.n. immunization with CLH001 and PBK001 vaccines using the C57BL/6 mouse model. We observed the generation of strong humoral and cellular immune responses to both vaccines, including development of mucosal IgA and Th1/Th17 memory cells in the lung compartment. Interestingly, the CLH001 vaccine generated a stronger IgG humoral response while PBK001 generated stronger CD4^+^ T-cell memory. Boolean analysis of multiparameter flow cytometry further demonstrated a marked increase in mono- and polyfunctional cytokine-producing T cells following exposure to *Bpm* antigens. Importantly though, the development of greater polyfunctional CD4^+^ T cells and IL-17 effector function was also associated with moderate inflammatory pathology in lung of mice vaccinated with PBK001. Overall, we demonstrated that vaccine-induced protection by intranasal immunization with these *Burkholderia* Δ*tonB* Δ*hcp1* vaccines strongly correlated with both humoral and cellular immunity.

## Results

### Vaccination with CLH001 and PBK001 induces overlapping and unique *Bpm*-specific antibody responses

**Fig. 1:**
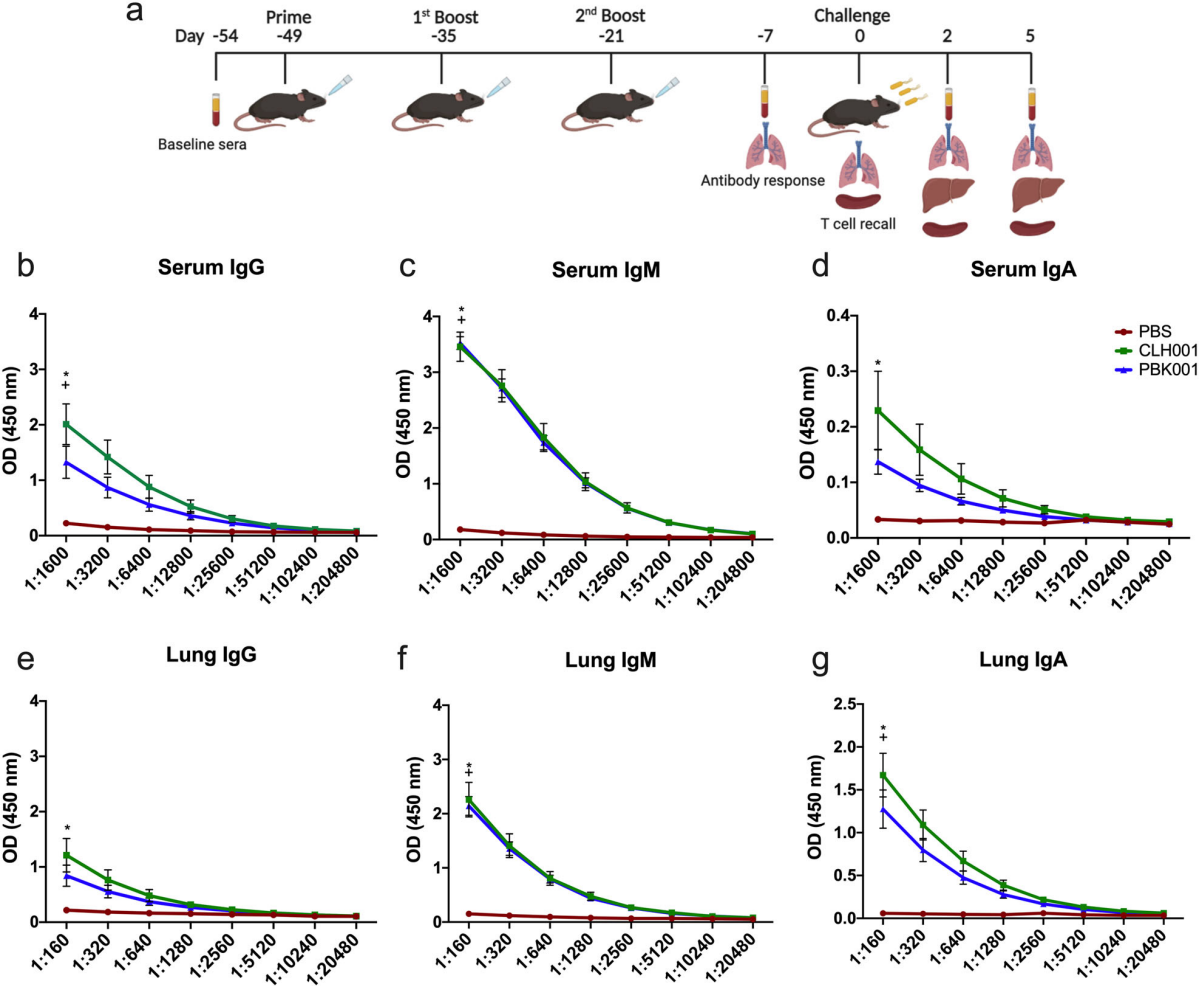
*Bpm*-specific antibody responses induced by CLH001 and PBK001 vaccination. **a** Experimental design and timeline of prime and boost regimen of PBS, CLH001, and PBK001 vaccination (i.n.), aerosol challenge and sample collection. **b** Serum anti-*Bpm* IgG antibody, **c** serum anti-*Bpm* IgM antibody, **d** serum anti-*Bpm* IgA antibody, **e** lung anti-*Bpm* IgG antibody, **f** lung anti-*Bpm* IgM antibody, **g** lung anti-*Bpm* IgA antibody. The sera (*n* = 10/group) and lung homogenate supernatant (*n* = 10/group) were collected two weeks after the 2^nd^ boost. ELISA was performed by incubating diluted serum and lung homogenate supernatants (2-fold dilution) with irradiated *Bpm* K96243 whole cell lysates. The graphs represent mean ± SEM of OD_450_ value. Data were analyzed using one-way ANOVA followed by Tukey test for multiple comparison. *p < 0.05 PBS vs. CLH001; ^+^*p* < 0.05 PBS vs. PBK001. The figure **a** was created using BioRender (https://biorender.com/).

To determine class and subclass of antibody responses against *Bpm* antigens in mice receiving PBS, CLH001, and PBK001, serum (*n* = 10/group) and lung homogenate (*n* = 10/group) were collected 2 weeks after the 2^nd^ boost (Fig. 1a). The quantitative results of OD (450 nm) values of antibodies binding to *Bpm* whole cell lysate were compared starting at a serum dilution of 1:1600 and lung homogenate dilution of 1:160. In the CLH001 and PBK001 vaccine groups, serum antibody specific to *Bpm* was predominantly IgM, followed by IgG and IgA (Fig. 1b–d). A different pattern of antibody class responses was observed in lungs. Compared to serum, a marked increase in pulmonary IgA was observed in lungs of mice vaccinated with either CLH001 or PBK001 (Fig. 1e–g). In contrast, both serum and lung of mice receiving PBS showed low responses in all antibody classes (Fig. 1b–g). The IgG subclass in serum of non-vaccinated and vaccinated mice was also evaluated in both vaccinated groups. IgG_2b_ and IgG_2c_ were shown to be the major subclasses of antibody specific to *Bpm* WCL as compared to IgG_1_ and IgG3 (Supplementary Fig. 1a–c).

### Immune serum from CLH001 and PBK001 immunized mice enhances in vitro bacterial killing and promotes opsonic activity of macrophages

**Fig. 2:**
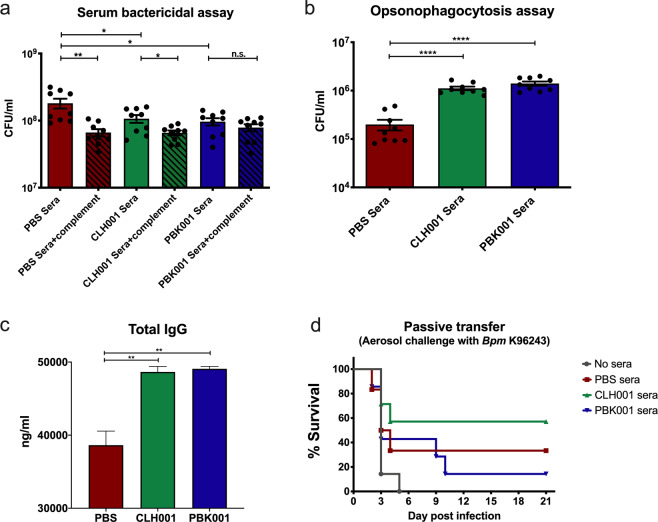
Serum from CLH001 and PBK001 immunized mice enhances bacterial killing and promotes opsonic macrophage activity but is insufficient to protect mice from *Bpm* infection. The immune serum from mice (*n* = 10) receiving PBS, CLH001, and PBK001 were collected two weeks after the 2^nd^ boost. **a** The serum bacterial killing assay was performed by incubating *Bpm* K96243 cells (1 × 10^6^ CFU) with 20% heat-inactivated (HI) pooled serum from non-vaccinated and vaccinated mice with and without complement. CFU/ml was determined after 4 h of incubation. **b** Opsonophagocytosis assay was performed by incubating the HI pooled serum with *Bpm* K96243 (5 × 10^5^ CFU) for 1 h. Opsonized bacteria were added to RAW 264.7 cells monolayers (5 × 10^5^ cells/well). Uptake was quantitated at 3 h post infection. Values of serum bacterial killing and opsonophagocytosis assay are represented as mean ± SEM from three individual assays conducted in triplicate. **c** Total IgG of post-vaccine sera used for passive transfer was quantitated using ELISA. Data are represented as mean ± SEM of triplicate assay. **d** Passive transfer of immunized and non-immunized mice was evaluated by i.p. injection of pooled serum (500 μl) to naïve C57BL/6 mice (*n* = 6–7/group). Two hours after serum transfer, mice were aerosol challenged with 18–30 LD_50_ of *Bpm* K96243. Survival of mice from PBS, CLH001, and PBK001 groups were observed for 21 days. *p < 0.05, **p < 0. 01, ***p < 0.001, ****p < 0.0001, n.s. = not significant. *P* values were determined using unpaired *t*-tests.

To determine whether antibodies generated by vaccination were directly bactericidal to *Bpm*, a suspension of mid-log phase of *Bpm* was incubated in 20% HI serum from CLH001- and PBK001-vaccinated mice in the presence or absence of complement from baseline sera. The results showed that incubation of serum from CLH001 and PBK001 vaccinated mice resulted in enhanced bactericidal activity in the absence of complement compared to serum from PBS-vaccinated mice (Fig. 2a). When complement was added, there was no difference in bacterial killing following exposure to serum from CLH001- and PBK001-vaccinated mice compared to naïve serum (Fig. 2a).

The preincubation of HI serum from both CLH001 and PBK001 vaccinated groups significantly enhanced bacterial uptake by RAW 264.7 murine macrophage cells as compared to the responses observed with PBS serum (Fig. 2b). Taken together, these results demonstrated that both vaccines induced similar production of specific antibodies with both opsonizing and direct bactericidal activity against *Bpm*.

### Passive transfer of serum from immunized mice does not protect mice against *Bpm* aerosol challenge

To determine whether serum from immunized mice could provide protection against *Bpm* infection, naïve C57BL/6 recipient mice were passively transferred with individual pooled serum from non-vaccinated (control), or CLH001- and PBK001-vaccinated mice. The total IgG of pooled post-vaccination serum was quantitated and compared among vaccination groups. Results showed significant increases of total concentration in post-vaccination serum from CLH001 (48,645 ng/ml) and PBK001 (49,075 ng/ml) compared to the non-vaccinated (PBS) group (38,630 ng/ml) (Fig. 2c). A passive transfer experiment was performed where mice received pooled serum through an i.p. injection with subsequent challenge with 18–30 LD_50_ of *Bpm* K96243 via the aerosol route. The serum from animal inoculated with PBS, CLH001, and PBK001 provided partial protection against *Bpm* challenge, with 33% (2 out of the 6 mice), 57% (4 out of the 7 mice), and 14% (1 out of the 7 mice) survival by day 21 post challenge, respectively, whereas all naïve mice died by day 5 post challenge (Fig. 2d). The modest increase in survival of mice receiving serum from CLH001-vaccinated animals did not reach significance compared to the other groups. These results indicate that antibodies provide only partial protection against *Bpm* challenge that includes non-specific innate antibody activity. Despite the significant increase in total and *Bpm*-specific antibody generated by CLH001 or PBK001 vaccination, the humoral immune components in the blood of immunized animals did not provide enhanced protection compared to control as a stand-alone correlate.

### Intranasal immunization with CLH001 and PBK001 generates spleen and lung populations with cytokine recall to *Bpm*

**Fig. 3:**
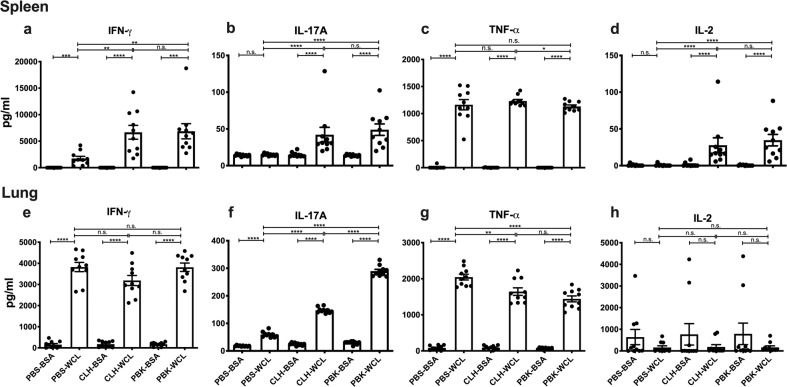
Respiratory immunization with CLH001 and PBK001 generates spleen and lung populations with cytokine recall to *Bpm*. C57BL/6 mice were inoculated with PBS (control), CLH001, or PBK001. Bone marrow-derived dendritic cells (BMDC) were pulsed with 1 μg WCL of *Bpm* K96243 or BSA (10 ug) as mock antigen. Spleen and lung were harvested at 21 days post vaccination, and mononuclear cells were isolated from disrupted organs. BMDC were overlaid with organ mononuclear cells and cultured at 37 °C and 5% CO_2_. Culture supernatants were harvested after 72 h co-culture with mock- or *Bpm-*pulsed BMDC. The production of cytokines in spleen (**a**–**d**) and lung (**e**–**h**) was measured by ELISA. Data are representative of mean ± SEM from 10 mice/group. Group comparisons were analyzed using *t*-test or Mann–Whitney test (*p < 0.05, **p < 0.01, ***p < 0.001, ****p < 0.0001, n.s. = not significant).

The cellular immune response associated with CLH001 and PBK001 vaccine-induced protection was examined on day 21 after the 2^nd^ boost. The lungs and spleens were collected from all vaccinated and control groups, and the cells were isolated, and co-cultured with BMDC pulsed with either non-specific control antigen (BSA), specific antigen (heat-killed *Bpm* K96243 WCL), or with T-cell receptor ligation positive control (CD3/CD28) for 72 h. The concentration of cytokines in spleen and lung supernatants were measured using ELISA (Fig. 3a–h). Levels of IL-17A and IL-2 that were activated by specific antigen in splenocytes of both CLH001 and PBK001 vaccinated groups were significantly higher than those for the PBS control group, which demonstrated negligible amounts of these molecules (Fig. 3b, d). Non-specific activation of IFN-γ was observed in PBS splenocytes activated with WCL; however, significantly greater activation was observed for CLH001 and PBK001 (Fig. 3a). The expression of TNF-α in spleen and lung of all three groups showed significant increases after stimulation with the WCL, reflecting the non-specific activation of diverse cell types including non-lymphocytic sources, such as macrophages (Fig. 3c, g). Non-specific activation of IFN-γ was also observed in lung cultures stimulated with *Bpm* WCL, while activation of IL-2 was not observed regardless of vaccine status (Fig. 3e, h). Lung IL-17 was marginally activated by antigen in cells from PBS vaccines and markedly increased in cells from the CLH001 or PBK001 groups (Fig. 3f). In contrast to the spleen, a further and significant increase in *Bpm*-specific IL-17A in the lung of mice vaccinated with PBK001 was observed compared to lung of mice vaccinated with CLH001 (Fig. 3f).

### CLH001 and PBK001 vaccines generate antigen-specific CD4^+^ T cells in the lung following intranasal immunization

**Fig. 4:**
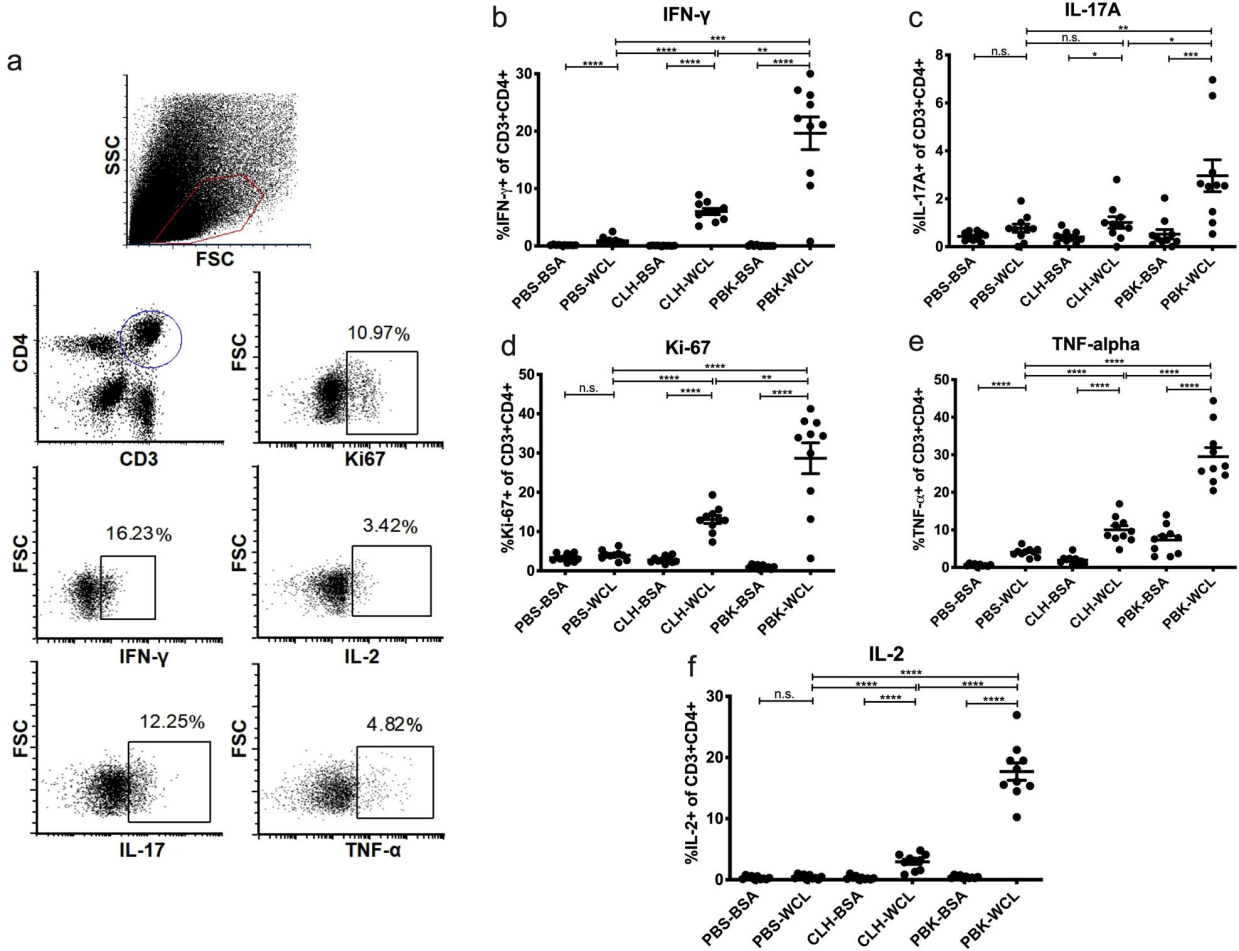
CLH001 and PBK001 vaccines generate antigen specific CD4^+^ T cells in the lung following intranasal immunization. At day 21 post vaccination, lungs of C57BL/6 mice receiving PBS, CLH001, or PBK001 were collected and disrupted cells were co-cultured with BSA (mock) or BMDC pulsed with 1 μg of heat-killed *Bpm* K96243 WCL. The cultured cells were harvested after incubation at 37 °C and 5% CO_2_ for 72 h, with addition of a Golgi protein transport inhibitor during the last 4 h of incubation. The markers were used to identify CD3 and CD4 T-cell populations and intracellular cytokines. **a** Representative gating strategy used to identify live cells with forward and side scatter properties of lymphocytes followed by selection of cells expressing the T lymphocyte markers CD3 and CD4. Intracellular cytokines including IFN-γ, TNF-α, IL-2, and IL-17 were further detected, along with the Ki-67 proliferation marker, following fixation and permeabilization of cells. Expression of intracellular cytokines IFN-γ (**b**), IL-17A (**c**), proliferation marker Ki-67 (**d**), TNF-α (**e**), and IL-2 (**f**) by CD3 + CD4^+^ T cells was determined in lungs of vaccinated and non-vaccinated mice. Data are represented as mean ± SEM from 10 mice/group. Significant values were analyzed using *t*-test or Mann–Whitney test (*p < 0.05, **p < 0.01, ***p < 0.001, ****p < 0.0001, n.s. = not significant).

Assessment of lung T-cell populations by flow cytometry demonstrate the development of antigen specific CD4^+^ T lymphocytes in lung of mice vaccinated with CLH001 and PBK001 (Fig. 4a–f). Activation with the Dynabead mouse T-cell activator CD3/CD28 positive control demonstrated that lung T-cell populations were responsive as indicated with activation of IFN-γ (0.41%), IL-17A (1.65%), Ki-67 (8.02%), TNF-α (13.7%), and IL-2 (6.9%) as a percent of total T cells. In response to *Bpm* WCL presentation by BMDC, lung CD4^+^ T cells from vaccinees showed significantly increased IFN-γ, IL-17A, TNF-α, and IL-2 cytokines in comparison to non-vaccinated mice or non-specific (BSA) antigen (Fig. 4b, c, e, f). The proliferation marker Ki-67 was significantly upregulated in lung CD4^+^ cells from both the vaccinated groups as compared to the PBS group (Fig. 4d), demonstrating the development of antigen-specific lymphoproliferation. The activation of cytokine and proliferative effector function by CD4^+^ T lymphocytes was especially marked in lung of PBK001 animals, an effect that reached significance for IFN-γ, IL-17A, Ki-67, TNF-α, and IL-2 responses as compared to PBS and CLH001 cells.

These results further demonstrated that the PBK001 vaccine induced a stronger antigen-specific cellular response, as indicated by greater production of effector cytokines characteristic of Th17 (IL-17A) and Th1 (IFN-γ) immune responses. Moderate background expression of IFN-γ and TNF-α was also observed in CD4^+^ T cells from lung of the PBS group (Fig. 4b, e), consistent with non-specific activation of cytokines in response to pathogen pattern recognition receptor ligation by *Bpm* WCL antigens. The numbers of lung CD8^+^ T cells were insufficient to develop adequate comparisons or establish statistical significance.

Examination of effector function of the splenic populations revealed a more moderate recall response to antigen as compared to lung. Antigen-specific differences in IFN-γ production by CD4^+^ T lymphocytes from vaccinated animals were evident (Supplementary Fig. 2a). Similar differences were not observed in the CD8^+^ T cells, although all three groups showed some level of expression (Supplementary Fig. 2f). No significant differences in activation of IL-17A and Ki-67 in either the CD4^+^ or CD8^+^ T-cell subsets were observed following antigen exposure (Supplementary Fig. 2b, e, g, j). Antigen-specific IL-2 was expressed by CD8^+^ T splenocytes from the PBK001 group whereas the differences in the CD4^+^ T cells among the groups were non-significant (Supplementary Fig. 2c, h). Surprisingly, antigen-specific TNF-α expression was more pronounced in splenic CD8^+^ T cells as compared to the CD4^+^ T-cell subset (Supplementary Fig. 2d, i) from both groups.

### PBK001 activates an increased and unique multifunctional CD4^+^ T-cell memory profile compared to CLH001

**Fig. 5:**
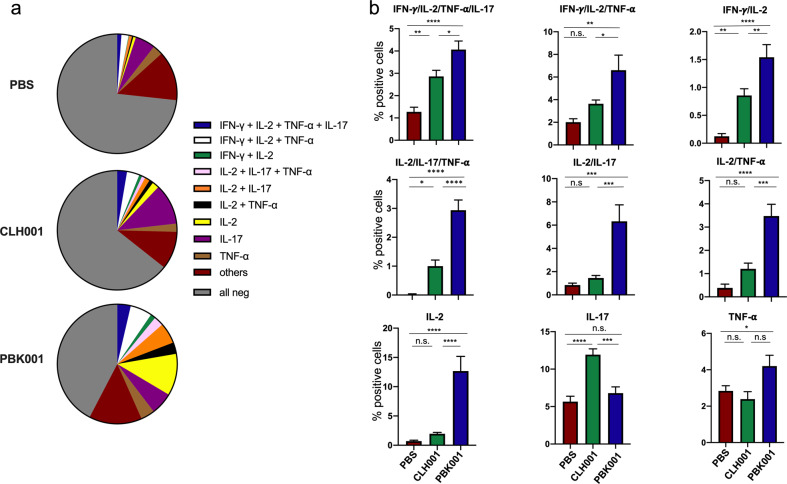
Development of antigen-specific and multi-functional CD4^+^ T-cell memory following vaccination. At day 21 post vaccination, lungs of C57BL/6 mice receiving PBS, CLH001, or PBK001 were collected and disrupted cells were co-cultured with BSA (mock) or BMDC pulsed with 1 μg of heat-killed *Bpm* K96243 WCL. The cultured cells were harvested after incubation at 37°C and 5% CO_2_ for 72 h, with addition of a Golgi protein transport inhibitor during the last 4 h of incubation. CD4^+^ T-cell recall was assessed by using flow cytometry to detect surface phenotype (CD3, CD4) and intracellular cytokines (IL-2, TNF-α, IFN-γ, and IL-17). Flow cytometry data were analyzed using FlowJO software including use of Boolean gate analysis to assess the frequency of multifunctional CD4^+^ T-cell populations. **a** Pie charts of background-subtracted Boolean gating analysis of selected mono-functional and poly-functional cytokine-producing CD4^+^ T cells following antigen-specific stimulation. **b** Comparisons among vaccine groups for polyfunctional outcomes. Pie slices in **a** represent the average, while bars in **b** represent the mean ± SEM of data from 10 mice pooled pairwise, in each vaccine group. Data are representative of three independent experiments with similar results. Remaining comparisons are shown in Supplemental Fig. 3. *p < 0.05, **p < 0.01, ***p < 0.001, ****p < 0.0001, n.s. = not significant.

Combinatorial cytokine expression in response to stimulation with specific antigen has been associated with development of optimum effector function by T cells. To further identify this important correlate of immunity, we assessed the multifunctionality of antigen-specific T cells in addition to the expansion of total cytokine-producing T-cell populations. Combinatorial Boolean gating analysis of data from multiparameter flow cytometry data was used to determine differences in multifunctional cytokine profiles using Flow Jo software. Numbers of CD4^+^ T cells in the lung were sufficient to perform this expanded assessment, while CD8^+^ T-cell numbers were insufficient. As illustrated in Fig. 5a, b, the endogenous responses, similar among PBS, CLH001, and PBK001 immunization groups, were characterized by cytokine-negative populations and populations expressing either IL-2 (IL-2+) or IL-17 (IL-17+) alone or in combination with TNF-α. Greater numbers of polyfunctional T cells were observed in the PBK001 vaccine group (53%) and the CLH001 group (31%) compared to the mock-vaccinated (PBS) group (24%) (Fig. 5a).

The overall intracellular cytokine recall response in PBK001-vaccinated mice was more diverse and polyfunctional in nature compared to CLH001-vaccinated mice (Fig. 5a). Populations expressing two to four cytokines were more abundant in lung of PBK001 as compared to CLH001, especially those expressing IL-2 or TNF-α as part of a multi-functional profile (Fig. 5b). Monofunctional CD4^+^ T-cell populations positive for TNF-α, and especially IL-2, were also more abundant in lung of PBK001 vaccinees following antigen exposure. In contrast, the CLH001 mice displayed a much more restricted phenotype, dominated by monofunctional expression of IL-17+ producing cells (Fig. 5b). Interestingly, IFN-γ was less frequently associated with polyfunctional profiles, generated by vaccination, compared to other cytokines (Supplementary Fig. 3).

### Intranasal immunization with CLH001 and PBK001 promotes a Th1 effector cytokine response and reduced bacterial burden upon *Bpm* infection

**Fig. 6:**
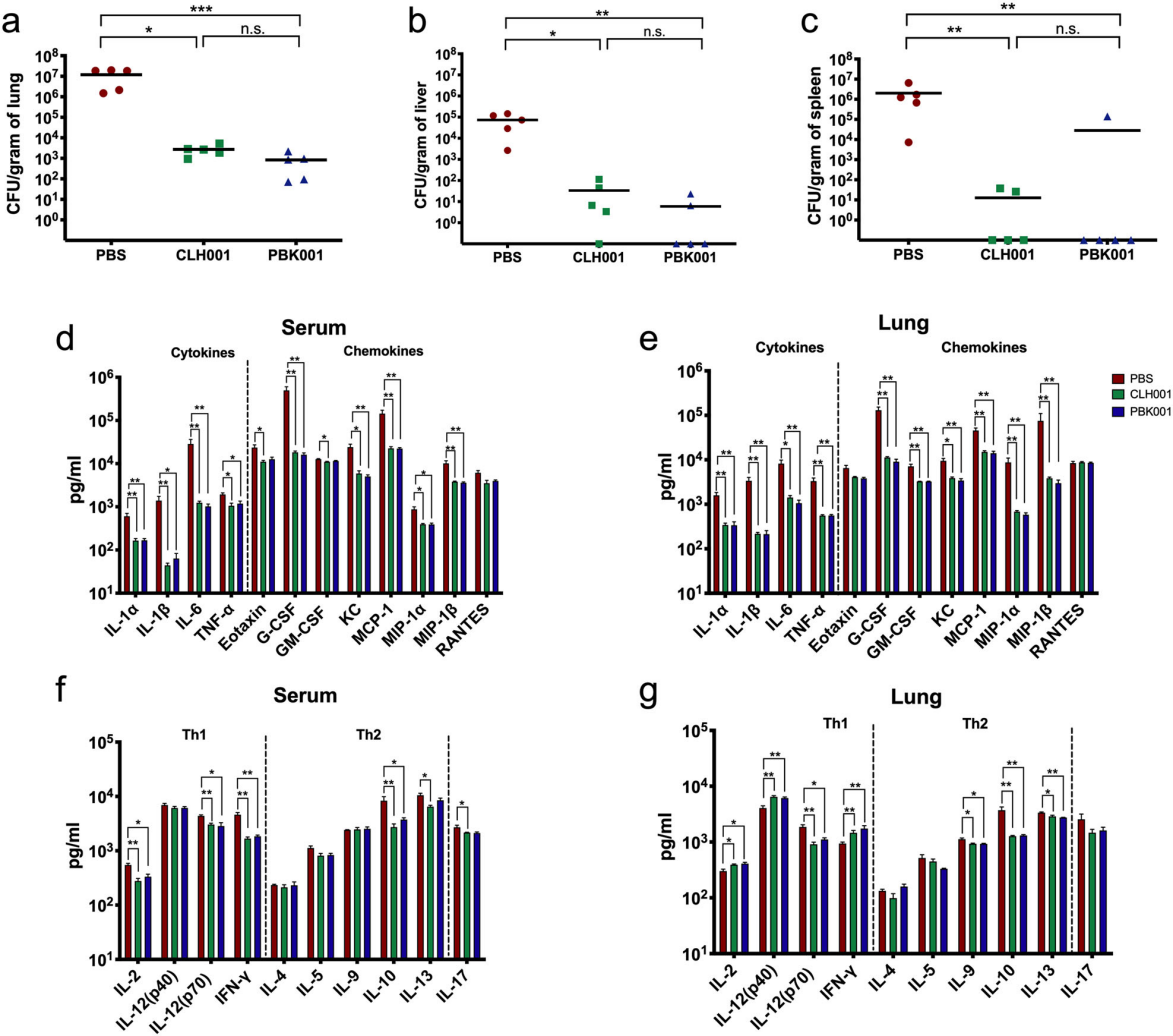
Intranasal immunization with CLH001 and PBK001 promotes a Th1 effector cytokine response and reduced bacterial burden upon *Bpm* infection. Bacterial burden in **a** lung, **b** liver, and **c** spleen of mice infected with *Bpm* K96243 were determined at 48 h after aerosol challenge. The symbols represent individual animals. The lines indicate the mean CFU/g of organ from each group. The proinflammatory cytokine/chemokine and Th1/Th2 effectors profiles in **d**, **f** serum and **e**, **g** lung of mice receiving PBS, CLH001, and PBK001 following 48 h exposure to *Bpm* K96243 via aerosol were measured using ELISA. Mean ± SEM plotted are representative of 5 animals. The significantly different values between non-vaccinated (PBS) and vaccinated groups were analyzed using *t*-test: *p < 0.05, **p < 0.01, ***p < 0.001, n.s. = not significant.

To understand the rapid immune response that occurred in immunized and challenged animals, we further examined the cytokines and chemokines in serum and lung supernatant as associated with bacterial burden at 48 h post challenge (Fig. 6). A significant reduction in bacterial burden in lungs, livers, and spleens of vaccinated mice was observed at 48 h post challenge, compared to control mice (Fig. 6a–c). Consistent with the reduced bacterial load in lung observed on day 2 upon aerosol challenge with *Bpm* K96243 (Fig. 6a–c), a significant down modulation of pro-inflammatory cytokines, IL-6, IL-1β, and TNF-α, were observed in serum (Fig. 6d) and lung (Fig. 6e) of mice from both vaccine groups as compared to the PBS group. Similarly, chemokines associated with bacterial proliferation and pro-inflammatory responses (e.g., G-CSF, KC, and MIP1-α) were also markedly reduced in vaccinated groups (Fig. 6d, e).

Overall, the post-challenge cytokine and chemokine signatures of the serum and lung reflected a reduced inflammation due to vaccine efficacy (Fig. 6d, e). Further, we observed the Th1 and Th2 effector cytokines in serum (Fig. 6f) and lung (Fig. 6g). We found the increased expression of IL-2, IL-12, and IFN-γ in lung of vaccinated mice (Fig. 6g). Thus, the vaccinated mice show considerable down modulation of cytokines and chemokines at challenge, and increased Th1 effector signature, that are associated with reduced immune pathology and bacterial load upon *Bpm* challenge.

### Lung and serum antibody responses differ between CLH001 and PBK001 vaccine groups post challenge

**Fig. 7:**
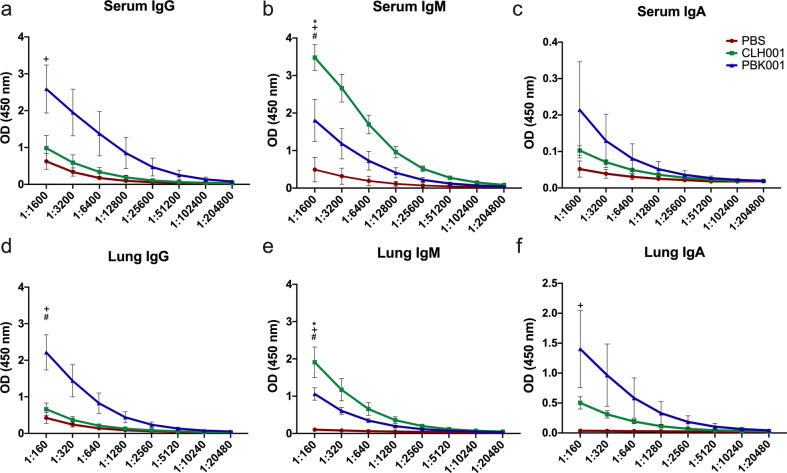
*Bpm*-specific serum and lung IgG and IgA are the predominant responses in PBK001-vaccinated mice during infection whereas IgM is a major response in CLH001. The serum and lung homogenate were collected from PBS-, CLH001- or PBK001-vaccinated mice after 48 h exposure to *Bpm* K96243 via aerosol route. The level of IgG (**a**, **d**), IgM (**b**, **e**), and IgA (**c**, **f**) was measured using ELISA by incubating diluted serum (*n* = 5/group) and lung homogenate supernatant (*n* = 5/group) (2-fold dilution) with irradiated *Bpm* K96243 whole cell lysate. The graphs represent mean ± SEM of OD_450_ value. Data were analyzed using one-way ANOVA followed by Tukey test for multiple comparison. *p < 0.05 PBS vs. CLH001; ^+^*p* < 0.05 PBS vs. PBK001; ^#^*p* < 0.05 CLH001 vs. PBK001.

Serum and lung homogenates of immunized and non-immunized mice (*n* = 5/group) were collected 48 h after aerosol challenge. The distribution of *Bpm*-specific IgG, IgM, and IgA antibody during infection was evaluated. The PBS-administered group displayed only moderate antibody responses compared to the vaccine groups as characterized by IgG > IgM > IgA. Serum and lung from CLH001-vaccinated mice demonstrated production of IgM > IgG > IgA (Fig. 7a–f) post challenge. In contrast, serum from PBK001-vaccinated mice demonstrated production of IgG > IgM > IgA while lung production was IgG > IgA > IgM. In comparison of responses between vaccine groups, we found that mice receiving the PBK001 vaccine had higher serum and lung IgG and IgA compared to mice receiving CLH001 (Fig. 7a, c, d, f). Interestingly, serum and lung IgM was the predominant response in mice vaccinated with CLH001 (Fig. 7b, e). Notably, the lung IgA level was about 7 times higher than serum IgA in both CLH001 and PBK001 vaccinees. In PBK001 vaccinees, IgG levels were also greater post challenge as compared to post vaccination. Among the IgG subclass antibodies, serum IgG_2b_ was the predominant isotype observed post challenge (Supplementary Fig. 1d–f).

### Analysis of histology indicates that CLH001 and PBK001 vaccination is associated with mild to moderate lung inflammation post vaccination

**Fig. 8:**
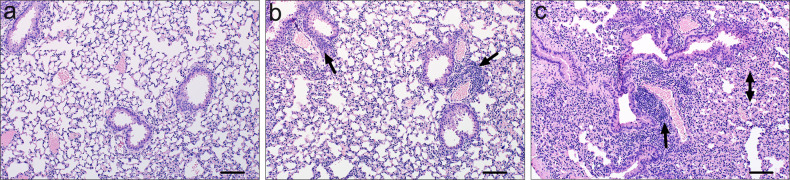
Analysis of histology indicates the CLH001 and PBK001 vaccination is associated with mild to moderate lung inflammation post vaccination. The lungs (*n* = 3/group) of mice receiving PBS (**a**), CLH001 (**b**), or PBK001 (**c**) were collected 2 weeks after the 2^nd^ boost. The lungs were placed in 10% formalin, fixed, paraffin-embedded, and stained with hematoxylin and eosin (H&E). The stained tissue sections were examined in a blind manner by a pathologist. The lungs of animals vaccinated with PBS was mostly non-remarkable except for some areas of mild interstitial inflammation. In lungs of CLH001 vaccinees, some areas of bronchovascular infiltrates (single arrow) were observed. The bronchovascular infiltrates were a more prevalent characteristic in lungs of mice vaccinated with PBK001, and frequent interstitial inflammation (double arrow) was also observed. Representative fields of view for lung tissues were taken at ×10 magnification. The scale bar represents 100 µm.

The lungs (*n* = 3/group) of mice receiving PBS, CLH001, or PBK001 were collected 2 weeks after the 2^nd^ boost. The stained lung tissues were examined in a blind manner by a pathologist. The lungs of animals vaccinated with PBS were mostly un-remarkable except for some areas of mild interstitial inflammation (Fig. 8a). In the lung of CLH001 vaccinees (Fig. 8b), some areas of bronchovascular infiltrates were observed. The bronchovascular infiltrates were a more prevalent characteristic in lungs of mice vaccinated with PBK001 (Fig. 8c), and frequent interstitial inflammation was also observed.

## Discussion

Multiple vaccine platforms against melioidosis are currently being developed because it is evident that the status of this disease remains an important public health concern, in addition to its potential as a biothreat agent^5^. Because *Bpm* is an intracellular bacterium, several studies have suggested that both humoral and cellular immunity are required to induce complete protection against infection^11^. The live attenuated vaccines are the most effective candidates providing strong and long-lasting immune response amid efforts to achieve immunity while mitigating the potential for wild-type pathogenic reversion. In previous studies, we employed a double gene deletion of *tonB* and *hcp1* to create an attenuated strain of *Bm* (CLH001) and *Bpm* (PBK001). The PBK001 vaccine conferred complete protection against *Bpm* wild-type challenge in C57BL/6 mice^13^ while 87.5% cross-protection was demonstrated with the CLH001 vaccine^14^. In contrast, CLH001 did not confer cross-protective immunity against *Bpm* in BALB/c mice^15^. In this study, we further evaluated the protective immunity generated by CLH001 and PBK001 vaccination against melioidosis in C57BL/6 mice.

Two weeks after the 2^nd^ boost, we found that CLH001 and PBK001 induced strong IgM, IgG_2_ (IgG_2b_ and IgG_2c_), and IgA antibody responses in serum and lung of C57BL/6 mice compared to PBS mock vaccination. These results demonstrate development of antibody classes that provide shorter term humoral immunity (IgM), as well as longer term responses such as IgG and IgA, that protect the periphery, tissue compartments, and mucosal sites. Surprisingly, we observed a complement-independent killing effect of immune serum from vaccinated mice. A similar outcome was observed in a study of serum generated by vaccination with the outer surface protein A (OspA) of *Borrelia burgdorferi*, a spirochete that causes Lyme disease^20,21^. The basis for these observed non-canonical functions of serum antibody to promote complement independent killing are not well defined, but could be attributed to catalytic activity, or direct inactivation of pathogens in the absence of effector cells and molecules^22,23^.

Moreover, the immune serum of both CLH001- and PBK001-vaccinated mice enhanced uptake of *Bpm* by macrophages. The specificity of IgM and IgG_2b_ responses has been shown against *Bpm* lipopolysaccharide (LPS) and capsule polysaccharide (CPS), respectively, in vaccinated BALB/c mice^24^. The result of our passive vaccination study using i.p. transfer of serum from vaccinated and mock-treated animals demonstrated a similar, and moderate, level of protection among all groups. It likely, then, that innate serum activity was an important contributor to the outcomes of our passive transfer experiments. Natural antibodies that are polyreactive against structurally diverse antigens have been described as an important innate antibacterial defense mechanism^25^. A review of passive transfer studies in *Burkholderia*, however, also demonstrates a variation of methodologies regarding the amount of serum used to transfer (50–1000 μl), the time between vaccination and challenge (1–24 h), and the different routes of challenge employed^18,26–29^. Therefore, passive transfer methodology of immune serum requires further evaluation.

Development of humoral immunity due to immunization was further evidenced by the class and subclass of antibody observed in mice after exposure to aerosolized *Bpm*. Both CLH001 and PBK001 vaccinees displayed significantly higher levels of IgG, IgM, and IgA antibody in serum and lung compared to PBS groups (Fig. 7) at 48 h post challenge. Among IgG subclasses, the IgG_2b_ subclass response was most prevalently observed following CLH001 and PBK001 vaccination (Supplementary Fig. 1). The detection IgA and IgG isotypes demonstrate that vaccination with these live vaccines generates robust, antigen-specific, B-cell activation and class switch recombination events that are important for humoral immune memory to *Bpm*.

The antigens recognized by antibody in animals vaccinated with CLH001 or PBK001, however, have not been defined to date. In other vaccine studies, development of serum antibodies against *Bpm* LPS, CPS, and flagellin correlated with protection in animal models of melioidosis^24,30,31^. Generation of IgG_2b_ responses following vaccination with *Bpm* CPS was also associated with protection in BALB/c mice^24^. In humans, survival in those who developed melioidosis correlated with high serum antibody (IgG) specific to LPS, but not CPS or flagellin^32^. These results are consistent with the generation of human serum IgG_2_ antibodies in response to bacterial polysaccharide antigens^33,34^.

Important differences in the humoral immune response of mice vaccinated with PBK001 and CLH001 were also observed in response to infectious challenge. A predominant IgM response was observed in serum and lung of CLH001 vaccines that was similar after challenge and vaccination. In contrast, a greater anamnestic IgG response was observed in serum and lung at challenge in mice vaccinated with PBK001. This outcome suggests that PBK001 immunization induced greater differentiation of IgG+ memory B cells, likely increasing the circulating and memory B cells in serum and lung, respectively. The presence of *Bpm*-specific IgA in the lung (Fig. 1), and not serum, of vaccinated mice indicated that i.n. immunization with CLH001 and PBK001 could induce a mucosal immune response in the murine respiratory system. Furthermore, a robust antigen-specific IgA response was detected in lung of vaccinated mice during *Bpm* infection (Fig. 7). This result indicates that IgA antibody may be critical for protecting mucosal surfaces from bacterial infection by direct neutralization or preventing binding to the mucosal surface^34^.

Antigen-specific T cells which produce multiple effector functions simultaneously, such as IL-2 and IFN-γ, have been shown to be a hallmark of protective immunity in controlled viral infections, such as cytomegalovirus (CMV) and Epstein-Barr virus (EBV)^35,36^. Prime-boost vaccine strategies that can generate polyfunctional CD4^+^ T-cell memory responses against other pathogens such as *Mycobacterium tuberculosis*^37^ have been shown to confer long-lasting protection against infection. To date, the effector profile that defines an optimal T-cell response against *Burkholderia* spp. has not been described. However, several studies have associated polyfunctional CD4^+^ T cells that express IL-2 and TNF-α, or that express IL-2, TNF-α, and IFN-γ, with increased bacterial containment^38,39^. Consistent with these reports, our findings demonstrate a strong association of the polyfunctionality of the antigen-specific T cells from the lung with vaccine protection. We observed both greater numbers and a more diverse repertoire of polyfunctional T cells after immunization with the highly protective PBK001 vaccine.

Monofunctional CD4^+^ T cells expressing IL-2 or TNF-α were the predominant cytokines in the recall response in lung of PBK001 animals, while monofunctional IL-17^+^ cells predominated in the CLH001 response. However, IL-17 was frequent among cytokines expressed by multiple polyfunctional populations that were significantly more frequent in lungs of PBK001-vaccinated mice. The classic Th1 cytokine IFN-γ was among the polyfunctional profile observed in many cells, although surprisingly less frequent than IL-2, TNFα, or IL-17. Plasticity and complementation of the Th1/Th17 axis is thought to be advantageous in an inflammatory environment, to maintain a flexible T-cell response capable of maintaining bacterial control while minimizing lung pathology. Early production of IL-17 promotes recruitment of Th1 cells, and in contrast IFN-γ can regulate the potentially damaging Th17 responses that occur in chronic stages of infection in diseases such as tuberculosis^40–43^. We did not observe significant development of the IL-17/IFN-γ dual-expressing cells that have been shown to activate nitric oxide synthase and correlate with vaccine-induced protection against pathogens, such as *Chlamydia muridarum*^44,45^, suggesting unique effector profiles directing pathogen-specific memory.

Surprisingly, the marked increase in IL-17 and IL-17-producing cells observed in the antigen-specific recall response, following PBK001 vaccination, did not correspond to a significant increase in protection in our study. It should be noted that in prior studies, we have some evidence that PBK001 has greater efficacy compared to CLH001. Importantly, we do observe evidence for moderate inflammatory responses post vaccination of the lung from PBK001 animals; an outcome known to occur through IL-17-mediated mechanisms such as neutrophilia. These results indicate the need to further improve a balance between protection and pathology during vaccine development and testing strategies in which Th17 memory cell differentiation is required. Vaccination with either CLH001 or PBK001, however, reduced the pro-inflammatory response to infectious challenge (Fig. 6). Cytokines and chemokines associated with inflammation and lung damage such as IL-1β, IL-6, and TNF-α were markedly reduced in lung and serum of vaccinees. In contrast, Th1 signature cytokines such as IL-2, IL-12, and IFN-γ were elevated in the lung of vaccinated mice. Overall, these results support the development of protective immune responses including memory T cells with effector function.

There is limited information on the nature of the protective immune response to *Bpm* infections in humans, but it is clear that both T- and B-cell-mediated responses are required^46^. Because *Bpm* is an intracellular pathogen, our previous studies of immunity in mice immunized with live attenuated mutants have suggested the role of T cells during infection^13^. The CD8^+^ T-cell responses that are developed following vaccination have been shown only marginally, at best, to contribute to protection against disease^18,47,48^. In contrast, studies in vaccinated mice as well as immune responses in humans in endemic areas have suggested that CD4^+^ T-cell responses play an important role^18,48–50^. Our data build upon these findings to further demonstrate the development of unique polyfunctional subpopulations that are associated with protection generated by the CLH001 and PBK001 vaccines.

Overall, our data suggest that vaccination with *Bm* CLH001 had a weaker level of immune response against *Bpm* antigens compared to *Bpm* PBK001. The PBK001 vaccine was able to induce a stronger response of CD4^+^ T-cell cytokines, greater numbers of proliferative and polyfunctional T cells, (Figs. 4 and 5) as well as generation of IgG memory B cells to control infection (Fig. 7). We previously demonstrated similar memory recall by splenic T cells of CLH001 vaccinees exposed to *Bpm* and *Bm* WCL^14^, suggesting that in vitro antigen availability is unlikely to be the basis for this observation. A contributing factor could be the greater breadth of immunogenic antigens identified for *Bpm* as compared to *Bm*. These *Bpm* antigens, such as the flagellar antigen FliC^51^, may promote stronger humoral and cellular immune responses after immunization with PBK001 compared to CLH001. The flagellin FliC has been shown to contain several peptides that encompass strong CD4^+^ T-cell epitopes^30,31,52–56^.

In conclusion, we demonstrated broad and robust immune responses that correlate with protection generated by i.n. vaccination with the *Burkholderia* Δ*tonB* Δ*hcp1* live attenuated vaccines. Our study highlighted the efficacy of CLH001 and PBK001 to elicit both humoral and cellular immunity, which are required for complete protection against *Bpm* infection. We further show that generation of diverse populations of multifunctional cytokine producing, and *Bpm*-specific, CD4^+^ T-cell populations in the lung is strongly correlated with protective immunity by attenuated *Burkholderia* vaccines. Finally, our results also indicate the potential importance of determining the optimum balance of CD4^+^ T-cell memory phenotypes (e.g., Th1/Th17 bias) that mediate protection in the absence of immune pathology.

## Methods

### Bacterial and growth conditions

*B*. *mallei* Δ*tonB* Δ*hcp1* (CLH001) was cultured from a freezer stock and plated on LB agar containing 4% glycerol (LBG) and supplemented with 200 μM FeSO_4_·7H_2_O for 72 h at 37°C. *B*. *pseudomallei* Δ*tonB* Δ*hcp1* (PBK001) was cultured from a freezer stock and plated on LB agar (0.5% NaCl) supplemented with 200 μM FeSO_4_·7H_2_O for 48 h at 37°C. The bacterial colonies were inoculated into 20 mL of LBG broth (for CLH001) or 20 mL of LB (0.5% NaCl) broth (for PBK001), and incubated for 16 h with agitation at 37°C. Wild-type *B*. *pseudomallei* K96243 was streaked on LBG agar plate and grown in 20 mL LBG broth at 37°C for 12 h. All the experiments were performed according to CDC Select Agent regulations.

### Animals

Female, 6–8-week-old C57BL/6 mice were obtained from the Jackson laboratories. All mice in this study were handled in strict accordance with the recommendations in the Guide for the Care and Use of Laboratory Animals of the National Institutes of Health. Mice were housed in microisolator cages under pathogen-free conditions, provided with rodent feed and water ad libitum, and maintained on a 12-h light cycle in an animal biosafety level 3 (ABSL3) laboratory. All experimental protocols were reviewed and approved by the Institutional Animal Care and Use Committee of the University of Texas Medical Branch (protocol 0503014E) and the Animal Care and Use Review Office of the Department of the Army.

### Vaccination and challenge

C57BL/6 mice were inoculated with a prime and two boosts regimen with either PBS (50 μl), CLH001 (1.5 × 10^5^ CFU) or PBK001 (1.5 × 10^4^ CFU), at two week intervals, using an intranasal route as diagramed in Fig. 1a. At 3 weeks post vaccination, mice were challenged with 4 × 10^7^ CFU/ml of aerosolized *Bpm* K96243 (nebulizer concentration). Briefly, the UTMB aerobiology facility utilized a Biaera AeroMP aerosol management platform housed within the IsoGARD class III glovebox, a nebulizer, a stainless-steel dilution/delivery line, a rodent exposure chamber, a relative humidity/temperature transducer, and an impinger. Mice were placed into nose-only restraint tubes, transferred to stainless steel boxes, and loaded into a rodent exposure chamber. Mice were exposed to bacteria via a three-jet nozzle collison nebulizer for 15 min. Nebulizers containing appropriate concentrations of *Bpm* K96243 in 10 ml of LB and samples collected from a SKC Biosampler containing 20 ml of LBG were diluted and plated to determine the presented dose (Dp). The LD_50_ of *Bpm* K96243 aerosol challenge (nose-only) in C57BL/6 was calculated based on previous study (1LD_50_ = 154 CFU) as described^57^. Animal weight and survival were monitored until the end of study.

### Detection of antibody responses post vaccination and post aerosol challenge

Serum and lung homogenates were collected from immunized mice at 2 weeks after the 2^nd^ boost and 48 h post aerosol challenge. High binding 96-well-plates (Corning, 9018) were coated and incubated with 1 µg/well (100 µl) of irradiated *Bpm* K96243 whole cell lysate (WCL) diluted with 1X Dulbecco’s Phosphate-Buffered Saline (1X DPBS) (Corning), then incubated overnight at 4 °C. Coated plates were washed twice with 200 µl /well of washing buffer (0.05% Tween-20, 1X DPBS), and non-specific binding was blocked by incubating with 250 µl/well of blocking buffer (0.1% Tween-20, 1% BSA, 1X DPBS) for 2 h. Serum and lung homogenates were diluted to 1:1600 and 1:160, respectively, in sample diluent (0.05% Tween-20, 0.5% BSA, 1X DPBS). Two-fold dilutions were performed before incubation of the samples with 1:5000 dilution of HRP-conjugated goat anti-mouse IgG (1036-05), IgG_1_ (1070-05), IgG_2b_ (1093-05), IgG_2c_ (1077-05), IgG_3_ (1103-05), IgM(1020-05), or IgA (1020-05) antibodies (Southern Biotech) for 2 h. Plates were washed three times before adding 100 µl of 3,3′,5,5′ tetramethylbenzidine (1X TMB) substrate (Invitrogen, 420156) and then incubated at room temperature (RT) for 15 min. The reaction was stopped by adding 50 µl of 2 N H_2_SO_4_, and the samples were immediately read at 450 and 570 nm using a microplate reader (Bio-Tek Epoch Microplate Spectrophotometer plate reader). The final OD_450-_OD_570_ was reported and presented graphically.

### Serum bactericidal assay

A 12 h culture of *Bpm* K96243 was diluted 1:100 in fresh LBG and grown to log phase (OD_600_ of 0.60). The bacteria were adjusted to 1 × 10^6^ CFU and incubated with 20% heat-inactivated (HI) (56 °C for 30 min) pooled serum from mice vaccinated with PBS, CLH001, or PBK001 with or without 20% baseline sera (source of complement) in LB broth with shaking at 200 rpm at 37 °C for 4 h. After incubation, 10-fold serial dilutions of each culture tube were plated on LBG agar and plates incubated at 37 °C for 48 h. Bacterial counts were reported as CFU/ml. Each experimental group was assayed in triplicate, and three independent experiments were performed.

### Opsonophagocytosis

An opsonophagocytosis assay was performed as described^58^. We used the murine macrophage cell line RAW264.7 (ATCC TIB-71), which was maintained in complete Dulbecco’s modified Eagle’s medium (cDMEM) (Gibco, 11965118) and supplemented with 10% HI fetal bovine serum (Gibco, 16140071), antibiotics (penicillin and streptomycin, Gibco, 10378016), sodium pyruvate (Gibco, 11360070), and non-essential amino acid (Gibco, 11140050), and grown at 37 °C under an atmosphere of 5% CO_2_. The RAW264.7 cells were resuspended in cDMEM and transferred into 24-well tissue culture plates (ThermoFisher, 172475) at a density of 5 × 10^5^ cells/well then incubated overnight. A 12 h culture of *Bpm* K96243 was adjusted to 5 × 10^5^ CFU/well in cDMEM (without antibiotic) containing PBS-, CLH001-, or PBK001-vaccinated mouse immune sera (pooled from 10 mice/group and heat inactivated for 30 min at 56 °C) then incubated at 37°C for 1 h. Opsonized bacterial suspension was added to pre-washed RAW264.7 monolayers prior to incubation at 37 °C. After 1 h, the monolayers were washed twice with Hanks’ balanced salts solution (HBSS) (Gibco, 14175095) to remove extracellular bacteria. Infected RAW 264.7 cells were incubated with medium containing 250 μg/ml kanamycin (Sigma, 10106801001) to prevent growth of residual extracellular bacteria. The infected cells were washed twice with HBSS and lysed with 0.2% Triton X-100 (Sigma, X100) after 3 h incubation. The lysate was serially diluted and plated onto LBG agar plates for CFU enumeration. Each experimental group was assayed in triplicate, and three independent experiments were performed.

### Passive transfer

Female C57BL/6 mice (6–8-week-old) (*n* = 10/group) were immunized by the prime-boost protocol with CLH001, PBK001 or PBS. Whole blood was collected by cardiac puncture two weeks after the 2^nd^ boost. Pooled baseline and post-vaccination sera were used to quantify total IgG antibody by using the mouse IgG total Ready-SET-Go (Invitrogen, 885040022) according to manufacturer’s recommendations. Five hundred microliters of pooled sera from donor mice were transferred to recipient female C57BL/6 J mice (6–8-week-old) (*n* = 6–7/group) by intraperitoneal injection. Two hours after serum transfer, recipient mice were challenged with 18–30 LD_50_ of *Bpm* K96243 via the aerosol route. The survival and weight of mice were monitored and recorded for 21 days after challenge.

### T-cell recall assessment using flow cytometry and ELISA

The study was performed using bone marrow-derived dendritic cells (BMDC) to provide an efficient antigen presenting cells (APC) for assessment of CD4^+^ and CD8^+^ T-cell recall following vaccination. Following removal of muscle and connective tissues, bones were carefully cut at the ends to expose the marrow which was flushed out using RPMI-1640 medium (Gibco, 2240089) with 10% FBS (Gibco, 16140071) (cRPMI). Bone marrow cells were cultured in RPMI 1640-10% FBS containing 2 mM β-mercaptoethanol (Sigma, M6250) with 100 U/ml recombinant mouse GM-CSF protein (R&D systems, 415-ML-020) and 50 U/ml recombinant mouse IL-4 (R&D Systems, 404-ML-050) in the absence of antibiotics. Fifty percent of the culture medium was removed and replenished every 3 days with recombinant cytokines. Non-adherent BMDCs were harvested on day 5 and plated at 1.5 × 10^5^ cells per well of a 48-well plate (ThermoFisher, 152640) and incubated in cRPMI without cytokines for another 24 h. BMDC were pulsed with 10 µg of BSA (negative control) (Sigma, A2153) or 1 µg of heat-killed *Bpm* K96243 whole cell lysate (WCL). Five hours following the pulse with control or specific antigen, BMDC were overlaid with isolated lung or spleen cells in ratio of 10:1 with BMDC for 72 h to assess antigen-specific T-cell recall. As a positive control for T-cell activation, the Dynabead mouse T activator CD3/CD28 (Thermo Fisher, 11452D) was used in place of BMDC.

Spleens and lungs were collected from vaccinated mice at day 21 after the 2^nd^ boost. For spleen, single-cell suspensions were isolated through a 100 μm nylon cell strainer (BD Falcon, 352360) then treated with 1X Red Blood Cell (RBC) lysis buffer (Invitrogen, 00-4300-54). Splenocytes were adjusted to a concentration of 1.5 × 10^6^ cells/ml (10:1) before seeding in a 24-well plate containing pulsed BMDC and incubated for 72 h. For lung cell isolation, organs were transferred to a petri dish and disrupted with sharp scissors prior to passage through a 100 μm nylon cell strainer (BD Falcon, 352360). Lung cell suspensions were centrifuged twice at 60 × *g* for 1 min at room temperature (RT), to remove fibroblasts and other large non-leukocytes, then supernatant was transferred to a new tube. The supernatant was centrifuged at 300 × *g* for 5 min and cell pellet resuspended in media. Lung cells were seeded 1.5 × 10^5^ cells/ml (1:1) in 48-well plate containing pulsed BMDC and incubated for 72 h.

Lung and spleen cells were harvested for flow cytometric analysis following 72 h of culture with BMDC. Golgi stop (BD Pharmingen, 554715) (1 μg/ml) was added 4 h prior to harvest to retain intracellular cytokines. Supernatants were collected and preserved for later cytokine measurements, and cells were used to assess T-cell memory recall. Briefly, flow cytometry was performed by re-suspending cells in 500 μl of FACS buffer (PBS supplemented with 1% HI FBS). Cells were stained with the Near IR live/dead viability marker (Thermo Fisher, L10119), followed by incubation with Fc block (BD Bioscience, 553142) to reduce non-specific binding of conjugated antibodies to the cells. Cellular phenotype was identified by incubating for 30 min with antibodies directed to surface markers, including CD3 (APC, eBioscience, 17-0032-82), CD4 (PE, eBioscience, 12-0042-85), and CD8 (PerCP Cy 5.5, eBioscience, 45-0081-82). Cells were washed with PBS, and subsequently fixed and permeabilized using the Cytofix-Cytoperm kit (BD Biosciences, 554715) as directed by the manufacturer. Following fixation, cells were washed in Permwash buffer (BD Biosciences, 554715) and incubated for 1 h with antibodies to intracellular molecules including interferon-γ (IFN-γ) (FITC, Biolegend, 505806), the proliferation marker Ki-67 (eFluor 450, eBioscience, 48-5698-82), interleukin-17A (IL-17A) (Brilliant Violet 785 Biolegend, 506928), tumor necrosis factor- α (TNF-α) (Brilliant Violet 711, Biolegend, 506349), and interleukin-2 (IL-2) (PE-Dazzle 594, Biolegend, 503840). Cells were washed and fixed in 4% ultra-pure formaldehyde (Polysciences, 50-00-0) for 48 h and finally placed in 2% formaldehyde prior to analysis.

Flow cytometric assessment of samples was performed by acquisition on a BD LSRII Fortessa flow cytometer (UTMB Flow Cytometry and Cell Sorting Facility) and analysis of total cells and intracellular cytokines performed with FCS Express 6 software (De Novo Software). Compensation matrices for spectral overlap in the multicolor panels were developed using the UltraComp compensation bead controls (Beckman Coulter, 01-2222-41). Forward and side scatter characteristics of leukocytes were used to isolate cells of interest, followed by additional exclusions of doublets and non-viable cells. Viable T-cell populations were further selected by CD3 marker positivity and sub-gated into CD4 and CD8 positive populations (Fig. 4a). Mono-functional and multifunctional analysis of cytokine producing T cells was performed using FlowJo software (Tree Star Inc., Ashland, OR). Boolean gate analysis was used to determine the frequency of different combinations of cytokine producing cell populations as affected by vaccination status and specific vaccine.

Culture supernatants of splenocytes and lung cells were collected and analyzed for IFN-γ (Invitrogen, 88-7314-22), IL-17A (Invitrogen, 88-7371-22), IL-2 (Invitrogen, 88-7024-22), and TNF-α (Invitrogen, 88-7324-22) production, using ELISA kits according to the manufacturer’s instructions.

### Analysis of serum and lung cytokine/chemokines production after aerosol challenge

Three weeks after the 2^nd^ boost, mice (*n* = 10/group) were challenged via aerosol with a Dp of 425–603 CFU (3–4 LD_50_) of *Bpm* K96243. After challenge (48 h) with *Bpm* K96243, whole blood was collected by cardiac puncture, and organs (lung, liver, and spleen) were harvested. The whole blood was centrifuged at 5000 × *g* at RT for 5 min after complete clotting. The serum was transferred to a new tube and stored at −80 °C. The lung, liver, and spleen were homogenized using tissue grinders (Covidien, 3505SA) and a 100 µl aliquot removed for serial dilution and growth on LBG agar for CFU enumeration. The rest of the homogenized lung tissue was centrifuged at 800 x g at 4 °C for 10 min, then supernatant was collected and stored at −80 °C. The serum and lung tissue supernatant samples were inactivated by γ-irradiation and verified for sterility. To define the chemokine/cytokine levels in serum and lung tissue homogenate, a murine bioplex ELISA kit (BioRad Bio-Plex Pro Mouse Cytokine 23-plex Assay, M60009RDPD) was used according to the manufacturer’s recommendations using serum samples (diluted 1:4) or undiluted lung supernatant. Target molecules included interleukin (IL)-1α, IL-1β, IL-2, IL-3, IL-4, IL-5, IL-6, IL-9, IL-10, IL-12 (p40), IL-12 (p70), IL-13, IL-17A, eotaxin, granulocyte–colony-stimulating factor (G-CSF), granulocyte–macrophage colony-stimulating factor (GM-CSF), IFN-γ, keratinocyte-derived chemokine (KC), Monocyte-chemotactic protein (MCP-1), macrophage inflammatory protein (MIP)-1α, MIP-1β, RANTES, and tumor necrosis factor (TNF-α). Data values represent the mean ± the SEM of five animals per treatment and per day. Out of range values above the asymptote of equation (> OOR) were set to the highest extrapolated value to provide a conservative estimate that allowed statistical analysis. A significant difference (*p* ≤ 0.05) in individual serum and lung tissue cytokine levels in PBS vs. CLH001-or PBK001-treated mice at 48 h was determined by a non-parametric test (Mann–Whitney test).

### Histopathologic analysis of lung following pulmonary immunization

The lungs (*n* = 3/group) of mice receiving PBS, CLH001, or PBK001 were collected at 2 weeks after the 2^nd^ boost to assess development of inflammatory responses to immunization. The lungs were preserved in 10% formalin and embedded in paraffin. The paraffin embedded lungs were sectioned at 5 µm and stained with hematoxylin and eosin (H&E) by the UTMB surgical pathology core facility to visualize tissue structures, cell morphology, and potential inflammatory infiltrates. Light microscopy was used to perform a treatment-blinded analysis of histopathology features associated with pulmonary exposure to PBS (mock) or live vaccines.

### Statistical analysis

All data were analyzed using GraphPad Prism version 8. To determine significance of intergroup difference for serum bactericidal assay, opsonophagocytosis assay, T-cell recall, total IgG, ELISAs of cell supernatant, bacterial burden, and cytokine/chemokine from Bioplex assay, the log-transform, and Student’s *t* test was used. The multifunctional T-cell memory and level of antibody response were analyzed by using one-way analysis of variance (one-way ANOVA). Comparisons among groups were further assessed using Tukey’s test. The survival curve of the passive transfer experiment was analyzed by using Log-rank (Mantel–Cox) test. *P*-values of ≤0.05 were considered as significant differences.

### Reporting summary

Further information on research design is available in the Nature Research Reporting Summary linked to this article.

## Supplementary information

Supplmentary Information

Reporting Summary

## Data Availability

All data generated or analyzed during this study are included in this published article and its supplementary files.
