# Supplementary material for: Antigen-specific antibody and polyfunctional T cells generated by respiratory immunization with protective BurkholderiaΔtonBΔhcp1 live attenuated vaccines
Source: NPJ Vaccines. 2021 May 13;6:72. doi: 10.1038/s41541-021-00333-4 (PMC8119421; doi:10.1038/s41541-021-00333-4)
Supplement: Supplementary file 1 — Supplmentary Information [file 41541_2021_333_MOESM1_ESM.pdf]

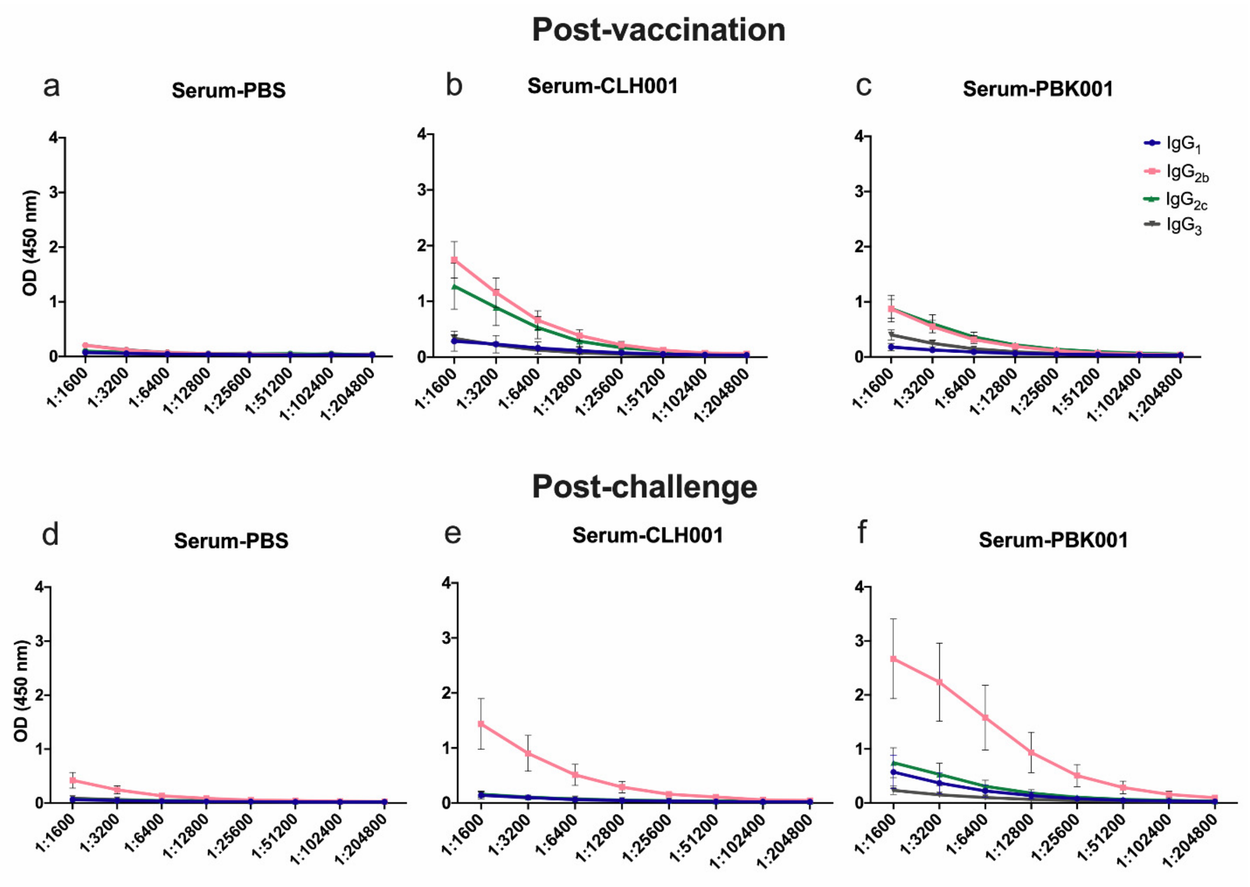

Supplementary Figure 1. CLH001 and PBK001 vaccination show different predominance of IgG subclass antibody responses post-vaccination and post-infection. The serum was collected from mice inoculated with PBS, CLH001 and PBK001 at two weeks after the 2nd boost (**a-c**) and 2 days post- challenge (**d-f**). The levels of IgG subclasses in serum were measured using ELISA. The linear graphs represent as mean ± SEM of OD_{450} value of immunoglobulin in diluted serum (n = 5-10/group) against irradiated *Bpm* K96243 whole-cell lysate.

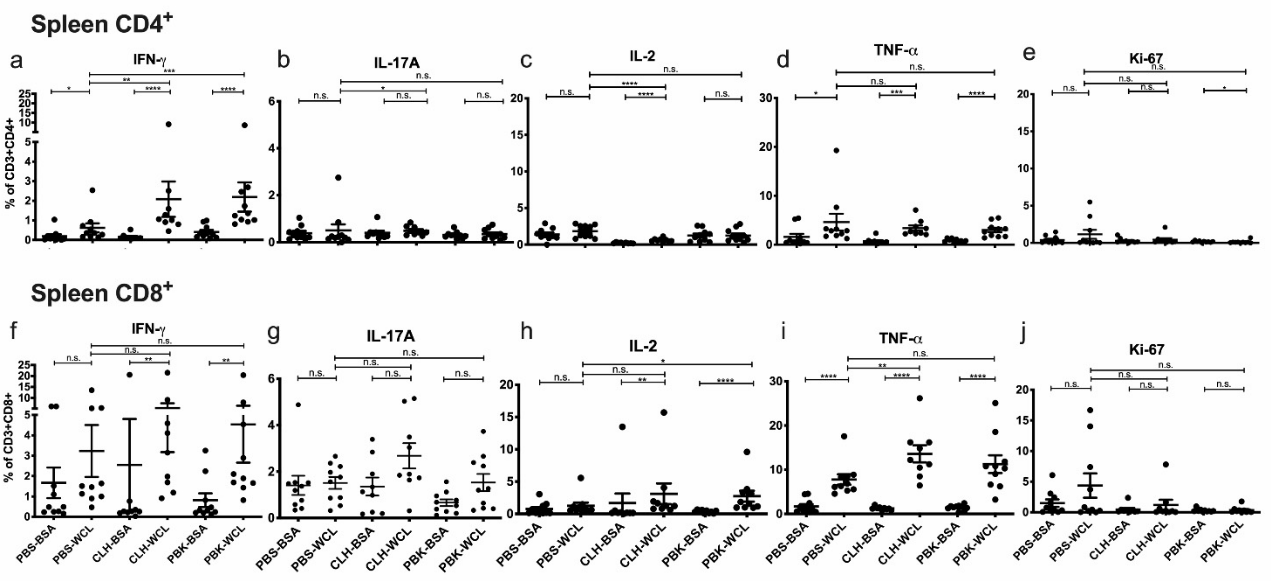

Supplementary Figure 2. Vaccination with CLH001 and PBK001 resulted in increased of IFN-γ CD4⁺ but no other intracellular cytokines in CD4⁺ or CD8⁺ T cells in spleen. At day 21 post-vaccination, spleens of C57BL/6 mice receiving PBS, CLH001 or PBK001 were collected and co-cultured with BSA (mock) or heat-killed *Bpm* K96243 WCL-pulsed BMDC. The cultured cells were harvested after incubation at 37°C and 5% CO_2 for 72 h. The cell surface markers were used to identify CD3, CD4 and CD8 T cell populations and intracellular cytokines. Expression of intracellular cytokines: IFN-γ (**a, f**), IL-17A (**b, g**), IL-2 (**c, h**), TNF-α (**d, i**) and proliferation marker Ki-67 (**e, j**) in spleens of vaccinated and non-vaccinated mice. Data are represented as mean ± SEM from 10 mice/group. Significant values were analyzed using *t*-test or Mann-Whitney test (*$p < 0.05$, **$p < 0.01$, ***$p < 0.001$, ****$p < 0.0001$, n.s. = not significant).

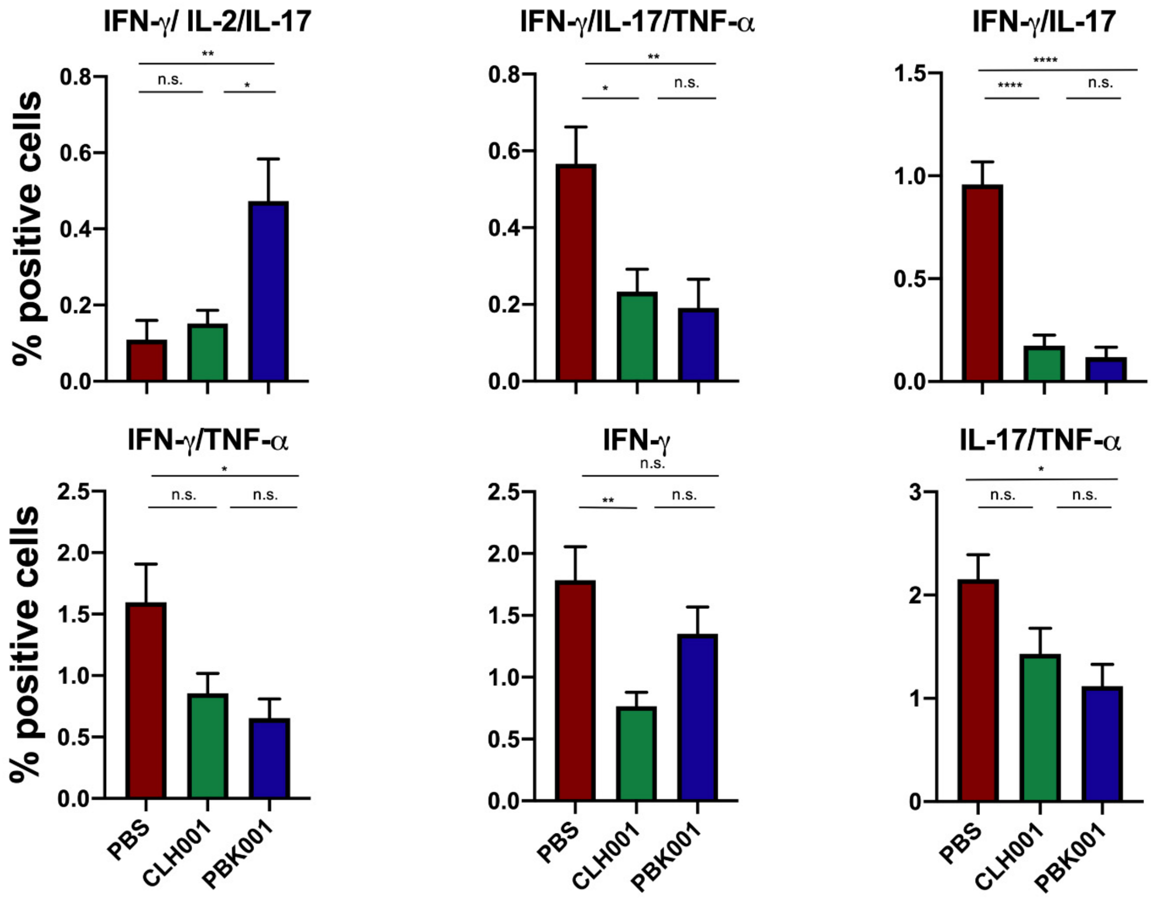

Supplementary Figure 3. Additional comparisons of post-vaccination development of antigen-specific and multi-functional CD4⁺ T cell memory in lungs. C57BL/6 mice were vaccinated with PBS (control), CLH001, or PBK001. Lungs were harvested at 21 days post vaccination and mononuclear cells were isolated from disrupted lung for antigen recall assays. Bone marrow-derived dendritic cells (BMDC) were pulsed with 1 µg whole cell lysate of *Bpm* K96243 or BSA (10 µg) as a mock antigen. BMDC were overlaid with lung mononuclear cells and cultured at 37°C and 5% CO_2 for 72 h with a Golgi transport inhibitor added during the last 5 h of culture to facilitate detection of intracellular cytokines. $CD4^+$ T cell recall was assessed by using flow cytometry to detect surface phenotype (CD3, CD4) and intracellular cytokines (IL-2, TNF-α, IFN-γ, and IL-17A). Flow cytometry data was analyzed using FlowJO software including use of Boolean gating to detect multifunctional $CD4^+$ T cell populations. Data are shown as the mean ± SEM of data from 10 mice in each vaccine group. The data were analyzed using one-way ANOVA followed by Tukey test for multiple comparison. (*$p < 0.05$, **$p < 0.01$, ****$p < 0.0001$**,** n.s. = not significant).
