# Supplementary material for: Antigen-specific antibody and polyfunctional T cells generated by respiratory immunization with protective BurkholderiaΔtonBΔhcp1 live attenuated vaccines
Source: NPJ Vaccines. 2021 May 13;6:72. doi: 10.1038/s41541-021-00333-4 (PMC8119421; doi:10.1038/s41541-021-00333-4)
Supplement: Supplementary file 2 — Reporting Summary [file 41541_2021_333_MOESM2_ESM.pdf]

nature research

Corresponding author(s): Janice Endsley, PhD

Last updated by author(s): Feb 9, 2021

Reporting Summary

Nature Research wishes to improve the reproducibility of the work that we publish. This form provides structure for consistency and transparency in reporting. For further information on Nature Research policies, see our Editorial Policies and the Editorial Policy Checklist.

Statistics

For all statistical analyses, confirm that the following items are present in the figure legend, table legend, main text, or Methods section.

n/a	Confirmed	
☐	☒	The exact sample size (*n*) for each experimental group/condition, given as a discrete number and unit of measurement
☐	☒	A statement on whether measurements were taken from distinct samples or whether the same sample was measured repeatedly
☐	☒	The statistical test(s) used AND whether they are one- or two-sided<br>*Only common tests should be described solely by name; describe more complex techniques in the Methods section.*
☒	☐	A description of all covariates tested
☐	☒	A description of any assumptions or corrections, such as tests of normality and adjustment for multiple comparisons
☐	☒	A full description of the statistical parameters including central tendency (e.g. means) or other basic estimates (e.g. regression coefficient) AND variation (e.g. standard deviation) or associated estimates of uncertainty (e.g. confidence intervals)
☐	☒	For null hypothesis testing, the test statistic (e.g. *F*, *t*, *r*) with confidence intervals, effect sizes, degrees of freedom and *P* value noted<br>*Give P values as exact values whenever suitable.*
☒	☐	For Bayesian analysis, information on the choice of priors and Markov chain Monte Carlo settings
☒	☐	For hierarchical and complex designs, identification of the appropriate level for tests and full reporting of outcomes
☒	☐	Estimates of effect sizes (e.g. Cohen's *d*, Pearson's *r*), indicating how they were calculated

Our web collection on statistics for biologists contains articles on many of the points above.

Software and code

Policy information about availability of computer code

Data collection	No specific code or software was utilized for the collection of data presented herein.
Data analysis	GraphPad Prism v8.0<br>FlowJO software<br>Boolean gating analysis

For manuscripts utilizing custom algorithms or software that are central to the research but not yet described in published literature, software must be made available to editors and reviewers. We strongly encourage code deposition in a community repository (e.g. GitHub). See the Nature Research guidelines for submitting code & software for further information.

Data

Policy information about availability of data

All manuscripts must include a data availability statement. This statement should provide the following information, where applicable:

- Accession codes, unique identifiers, or web links for publicly available datasets
- A list of figures that have associated raw data
- A description of any restrictions on data availability

All data generated or analysed during this study are included in this published article (and its supplementary information files).

Field-specific reporting

Please select the one below that is the best fit for your research. If you are not sure, read the appropriate sections before making your selection.

☒ Life sciences ☐ Behavioural & social sciences ☐ Ecological, evolutionary & environmental sciences

For a reference copy of the document with all sections, see nature.com/documents/nr-reporting-summary-flat.pdf

Life sciences study design

All studies must disclose on these points even when the disclosure is negative.

Sample size	No sample-size calculations were performed. Sample size was determined to be adequate based on the magnitude and consistency of measurable differences between groups.
Data exclusions	No data was excluded from the studies carried in this manuscript
Replication	All experimental replicates showed consistent results and appear in the manuscript
Randomization	All animal groups are randomly vaccinated and challenged when appropriate and care is taken to balance age of the mice used in the experiments.
Blinding	Experiments are appropriately carried to prevent any potential biased in different experimental groups.

Reporting for specific materials, systems and methods

We require information from authors about some types of materials, experimental systems and methods used in many studies. Here, indicate whether each material, system or method listed is relevant to your study. If you are not sure if a list item applies to your research, read the appropriate section before selecting a response.

Materials & experimental systems

n/a	Involved in the study	
☐	☒	Antibodies
☐	☒	Eukaryotic cell lines
☒	☐	Palaeontology and archaeology
☐	☒	Animals and other organisms
☒	☐	Human research participants
☒	☐	Clinical data
☐	☒	Dual use research of concern

Methods

n/a	Involved in the study	
☒	☐	ChIP-seq
☐	☒	Flow cytometry
☒	☐	MRI-based neuroimaging

Antibodies

Antibodies used	HRP-conjugated goat anti-mouse IgG (Southern Biotech™; Catalog No. 1036-05)<br>HRP-conjugated goat anti-mouse IgG1 (Southern Biotech™; Catalog No. 1070-05)<br>HRP-conjugated goat anti-mouse IgG2b (Southern Biotech™; Catalog No. 1093-05)<br>HRP-conjugated goat anti-mouse IgG2c (Southern Biotech™; Catalog No. 1077-146 05)<br>HRP-conjugated goat anti-mouse IgG3 (Southern Biotech™; Catalog No. 1103-05)<br>HRP-conjugated goat anti-mouse IgM (Southern Biotech™; Catalog No. 1020-05)<br>HRP-conjugated goat anti-mouse IgA (Southern Biotech™; Catalog No. 1020-05)<br>CD3 Monoclonal Antibody (17A2), APC (eBioscience™; ™ Catalog No.17-0032-82)<br>CD4 Monoclonal Antibody (RM4-5), PE (eBioscience™; Catalog No. 12-0042-85)<br>CD8a Monoclonal Antibody (53-6.7), PerCP-Cyanine5.5 CD8 (eBioscience™; Catalog No. 45-0081-82)<br>FITC anti-mouse IFN-γ Antibody (Biolegend; Catalog No. 505806)<br>Ki-67 Monoclonal Antibody (SolA15), eFluor 450 (eBioscience™; Catalog No. 48-5698-82)<br>Brilliant Violet 785™ anti-mouse IL-17A Antibody (Biolegend; Catalog No. 506928)<br>Brilliant Violet 711™ anti-mouse TNF-α Antibody (Biolegend; Catalog No. 506349)<br>PE/Dazzle™ 594 anti-mouse IL-2 Antibody (Biolegend; Catalog No.503840)
Validation	Commercial antibodies were used according to manufacturer's instruction and a statement of this is provided in the manuscript.

Eukaryotic cell lines

Policy information about cell lines

Cell line source(s)	RAW264.7 (ATCC TIB-71)
Authentication	Cell lines were not authenticated for the study but commercially-provided primary cells were given a certificate of authentication.
Mycoplasma contamination	Cell lines were not tested for mycoplasma. However, commercially-provided primary cells were given a certificate of authentication that they were negative for mycoplasma
Commonly misidentified lines (See ICLAC register)	No commonly misidentified cell lines were used in this study.

Animals and other organisms

Policy information about studies involving animals; ARRIVE guidelines recommended for reporting animal research

Laboratory animals	8-10 week old, female C57BL/6 mice were utilized in this study.
Wild animals	No wild animals were used in any study
Field-collected samples	No field-collected samples were used in this manuscript
Ethics oversight	All animal studies were previously approved

Note that full information on the approval of the study protocol must also be provided in the manuscript.

Dual use research of concern

Policy information about dual use research of concern

Hazards

Could the accidental, deliberate or reckless misuse of agents or technologies generated in the work, or the application of information presented in the manuscript, pose a threat to:

No	Yes	
☐	☒	Public health
☐	☒	National security
☐	☒	Crops and/or livestock
☐	☒	Ecosystems
☒	☐	Any other significant area

Hazards	Tier 1 Select Agent Burkholderia pseudomallei (Bpm) K96243

For examples of agents subject to oversight, see the United States Government Policy for Institutional Oversight of Life Sciences Dual Use Research of Concern.

Experiments of concern

Does the work involve any of these experiments of concern:

No	Yes	
☒	☐	Demonstrate how to render a vaccine ineffective
☒	☐	Confer resistance to therapeutically useful antibiotics or antiviral agents
☒	☐	Enhance the virulence of a pathogen or render a nonpathogen virulent
☒	☐	Increase transmissibility of a pathogen
☒	☐	Alter the host range of a pathogen
☒	☐	Enable evasion of diagnostic/detection modalities
☒	☐	Enable the weaponization of a biological agent or toxin
☒	☐	Any other potentially harmful combination of experiments and agents

Precautions and benefits

Biosecurity precautions	All manipulations of Bpm were conducted in CDC/USDA-approved and registered biosafety level 3 (BSL3) facilities at UTMB in accordance with approved BSL3 standard operating practices.

Biosecurity oversight	All experiments shown in this manuscripts were previously approved by Employee Health & Safety at UTMB in approved BSL3 and ABSL3 laboratories following CDC/USDSA-approved guidelines
Benefits	The results presented here show the potential of a pre-clinical vaccine candidate against Bpm in a rodent model of melioidosis. These results highlight the potential of this vaccine in mitigating the public health, agricultural, and/or potential national security threat that the pathogen Bpm poses in these areas.
Communication benefits	The benefits of the study presented show a potential vaccine candidate that protects against exposure to the Tier 1 Select Agent pathogen Bpm.

Flow Cytometry

Plots

Confirm that:

☒ The axis labels state the marker and fluorochrome used (e.g. CD4-FITC).

☒ The axis scales are clearly visible. Include numbers along axes only for bottom left plot of group (a 'group' is an analysis of identical markers).

☒ All plots are contour plots with outliers or pseudocolor plots.

☒ A numerical value for number of cells or percentage (with statistics) is provided.

Methodology

Sample preparation	*Describe the sample preparation, detailing the biological source of the cells and any tissue processing steps used.*
Instrument	*Identify the instrument used for data collection, specifying make and model number.*
Software	*Describe the software used to collect and analyze the flow cytometry data. For custom code that has been deposited into a community repository, provide accession details.*
Cell population abundance	*Describe the abundance of the relevant cell populations within post-sort fractions, providing details on the purity of the samples and how it was determined.*
Gating strategy	*Describe the gating strategy used for all relevant experiments, specifying the preliminary FSC/SSC gates of the starting cell population, indicating where boundaries between "positive" and "negative" staining cell populations are defined.*

☐ Tick this box to confirm that a figure exemplifying the gating strategy is provided in the Supplementary Information.
